# Phylogenetic Tree Instability After Taxon Addition: Empirical Frequency, Predictability, and Consequences For Online Inference

**DOI:** 10.1093/sysbio/syae059

**Published:** 2024-10-25

**Authors:** Lena Collienne, Mary Barker, Marc A Suchard, Frederick A Matsen

**Affiliations:** Computational Biology Program, Fred Hutchinson Cancer Research Center, 1100 Fairview Ave N, Seattle, WA 98109, USA; Computational Biology Program, Fred Hutchinson Cancer Research Center, 1100 Fairview Ave N, Seattle, WA 98109, USA; Howard Hughes Medical Institute, Fred Hutchinson Cancer Research Center, 1100 Fairview Ave N, Seattle, WA 98109, USA; Department of Human Genetics, University of California, 885 Tiverton Drive, Los Angeles, CA 90095, USA; Department of Computational Medicine, University of California, 885 Tiverton Drive, Los Angeles, CA 90095, USA; Department of Biostatistics, University of California, 650 Charles E. Young Dr. South, Los Angeles, CA 90095, USA; Computational Biology Program, Fred Hutchinson Cancer Research Center, 1100 Fairview Ave N, Seattle, WA 98109, USA; Howard Hughes Medical Institute, Fred Hutchinson Cancer Research Center, 1100 Fairview Ave N, Seattle, WA 98109, USA; Department of Statistics, University of Washington, Padelford Hall, Northeast Stevens Way, Seattle, WA 98195, USA; Department of Genome Sciences, University of Washington, 3720 15th Ave NE, Seattle, WA 98195, USA

**Keywords:** Maximum Likelihood, online phylogenetics, phylogenetic stability, taxon addition

## Abstract

Online phylogenetic inference methods add sequentially arriving sequences to an inferred phylogeny without the need to recompute the entire tree from scratch. Some online method implementations exist already, but there remains concern that additional sequences may change the topological relationship among the original set of taxa. We call such a change in tree topology a lack of stability for the inferred tree. In this article, we analyze the stability of single taxon addition in a Maximum Likelihood framework across 1000 empirical datasets. We find that instability occurs in almost 90% of our examples, although observed topological differences do not always reach significance under the approximately unbiased (AU) test. Changes in tree topology after addition of a taxon rarely occur close to its attachment location, and are more frequently observed in more distant tree locations carrying low bootstrap support. To investigate whether instability is predictable, we hypothesize sources of instability and design summary statistics addressing these hypotheses. Using these summary statistics as input features for machine learning under random forests, we are able to predict instability and can identify the most influential features. In summary, it does not appear that a strict insertion-only online inference method will deliver globally optimal trees, although relaxing insertion strictness by allowing for a small number of final tree rearrangements or accepting slightly suboptimal solutions appears feasible.

New sequence data are added to public databases at a high frequency. Adding new sequences to an already inferred phylogeny could reveal the relationship of this new taxon to the already analyzed taxa and could also be helpful in clarifying evolutionary relationships in the already existing phylogeny (Pollock et al. 2002). As new sequences get sampled over time, datasets keep growing in size. This requires methods to analyze new data while utilizing information received from previously analyzed data, for example, for automated species delimitation (Zhang et al. 2013). One approach to handle these scenarios is to develop “online” phylogenetic algorithms in which one can update an existing phylogenetic inference with additional sequences without running the analysis from scratch.

There has been considerable interest in and development of online methods for phylogenetics. Online methods are already available for Bayesian software packages BEAST and BEAST2 that employ Markov chain Monte Carlo (MCMC) sampling, and decrease MCMC chain convergence time but still require continued sampling after insertion of sequences (Gill et al. 2020; Bouckaert et al. 2022). An alternative for online Bayesian inference is sequential Monte Carlomethods (Dinh et al. 2018; Fourment et al. 2018), which hold promise to be more efficient than MCMC while being similarly accurate if path degeneracy issues are resolved (Fourment et al. 2018; Truszkowski et al. 2023). For maximum parsimony inference, online algorithms update the tree to optimize the parsimony score after inserting taxa in an already inferred tree (Ye et al. 2022). This strategy of re-optimization after insertion performs well for viral datasets. The resulting trees are comparable with Maximum Likelihood methods that compute trees from scratch while needing much less computational time (Kramer et al. 2023). The strategy of sequentially adding sequences to a tree and then updating it has been used to scale up Maximum Likelihood inference (Izquierdo-Carrasco et al. 2014; De Maio et al. 2023), and shows good performance for viral datasets (De Maio et al. 2023).

However, there has neither been a broad empirical study of how trees change when a taxon is added to a data set nor has there been work researching the factors determining whether such change happens. We call an inference “stable” upon taxon addition if the tree inferred on the alignment including the new taxon, contains the tree inferred for the alignment without the sequence of this taxon. As a similar concept to instability, “rogue taxa” have been studied in the literature, which are taxa that have an influence on the inferred tree in that they increase uncertainty in the inferred tree by lowering bootstrap support (Baurain et al. 2007; Trautwein et al. 2011). This concept of instability is different from the type of instability that we are interested in, which is the change of tree topology upon taxon addition. Although updating trees after the addition of new taxa is the central principle of most existing online algorithms, there has not been extensive research in assessing whether these updates are always required, and if they are, whether updates might only be needed in specific regions of a tree. Predicting instability in an efficient manner benefits the development of online algorithms, as predictions could be used to decide if a tree needs updating upon taxon addition.

This article aims to investigate the stability of taxon addition for Maximum Likelihood tree inference on empirical data. We analyze instability in a range of empirical datasets to establish whether instability as found in some cases in the literature (Mariadassou et al. 2012; Denton and Goolsby 2018; Bouckaert et al. 2022) occurs frequently or only in isolated cases. Our datasets comprise diverse sequence alignments containing gene sequences for a variety of species and have previously been used to study the performance of Bayesian phylogenetic inference (Harrington et al. 2021). Though we cover a variety of alignments with different properties, other types of data, for example, viral datasets, might show different behavior.

With our stability analysis, we aim to understand the following questions about stability:

- How frequent is instability in a large collection of empirical data sets?
- When instability is present, is the fully optimized tree significantly better according to statistical criteria?
- When instability is present, how far do changes propagate across the tree?
- Can we efficiently predict instability “ahead of time” via classification/regression using a battery of summary statistics?

To address these questions, we build a pipeline for analyzing empirical data to determine whether it is possible to predict stability. This pipeline is not designed for general use to predict the stability of a dataset, but rather to assess whether it is possible to predict stability at all.

We find that instability occurs frequently in our data examples, but is often not significant. When instability occurs, the changes in tree topology are usually not local to the insertion location of the new taxon. Instead, we often see instability in regions of low bootstrap support. Random forest regression and classification are capable of predicting stability for the datasets and summary statistics we consider with high correlation ($R^{2}=0.98$) and discrimination (Area Under the Receiver Operating Characteristic =0.93), revealing that insertion locations in regions of high uncertainty and non-tree-like evolution of the added taxon are the main drivers of instability.

## Methods

### Measuring Stability

**Figure 1:**
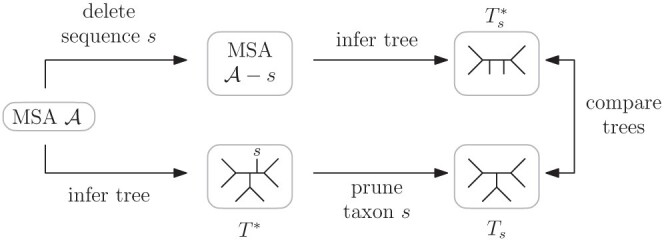
Determining whether an inference is stable for the addition of taxon s by comparing inferred tree $T_{s}^{*}$ and pruned tree $T_{s}$. When $T_{s}^{*}$ and $T_{s}$ are different, we say that the tree $T_{s}^{*}$ is *unstable* to the addition of s.

A tree inference on an alignment is stable for the addition of a taxon s if the tree inferred on the alignment without taxon s is contained in the tree inferred on the full alignment containing the sequence of taxon s (Fig. 1). In this article, we consider unrooted binary trees.

#### Taxon Influence Index

We used the taxon influence index (TII) (Mariadassou et al. 2012) to measure instability. Let the “full tree” $T^{*}$ be the tree inferred on an alignment 𝒜 and let 𝒜−s be this alignment with the sequence of taxon s removed. Then TII⁡(s) is defined as the distance between the “inferred tree” $T_{s}^{*}$, which is the tree inferred on 𝒜−s, and the “pruned tree” $T_{s}$, which results from pruning taxon s from $T^{*}$. All these trees are unrooted. We used the Robinson-Foulds (Robinson and Foulds 1981) (RF) distance to compute the TII:


$$\mathrm{TII}\left(s\right)=d_{\mathrm{RF}}\,\left(T_{s},T_{s}^{*}\right).$$

If the tree topologies of the 2 trees coincide, that is, their RF distance is 0, we say that the inference is stable, otherwise it is unstable. To generate a large dataset, we calculated the TII for every taxon of every alignment we included in our analysis. To normalize the TII, we divided it by 2⁢(n−3), which is the maximum possible RF distance between any two trees on n leaves.

#### Locations of Instability

We wished to measure how far from the location at which the additional taxon is attached changes have propagated, which we quantified with a metric we call the “disruption radius.” The disruption radius is the maximum distance between the attachment location of the added taxon s in the full tree $T^{*}$ and any edge of $T^{*}$ that is not present in $T_{s}^{*}$. Here, we used the topological distance, that is, the number of nodes between the edge incident to s and its most distant edge that is present in $T^{*}$ but not in $T_{s}^{*}$. Because the inferred tree $T_{s}^{*}$ does not contain taxon s, we considered an edge of the full tree $T^{*}$ to be present in the inferred tree $T_{s}^{*}$ if removing s from the split induced by this edge gives a split that is induced by an edge in $T_{s}^{*}$. If the inferred tree $T_{s}^{*}$ is identical to the pruned tree $T_{s}$, no edges differ between these trees and the disruption radius is 0, as is TII⁡(s). We normalized the disruption radius by dividing by the maximum distance of any internal edge to the edge incident to s in $T^{*}$.

We were also interested in the relationship between bootstrap support and unstable edges and hypothesized that instability is more likely to occur in areas of low bootstrap support. Therefore, we computed the average distance of edges that are in the inferred tree but not the pruned tree to their closest low bootstrap support (<70%) edge. For this, we used the normalized topological distance, which is the number of nodes between these edges, divided by the maximum distance of the given edge to any internal edge.

#### Testing Significance of Instability

To determine whether observed instability in the form of non-zero TII is significant, we performed the AU test (Shimodaira 2002). The AU test takes as input a list of trees, and for each tree tests the null hypothesis that this tree is equally good or better than all other trees in the input set. It uses bootstrapping to compute for each bootstrap sample the distribution of differences in log likelihood between the maximum likelihood tree and all other given trees. The bootstrap samples are used as a null distribution to compute a *P* value, and the null hypothesis is rejected if the *P* value is less than 0.05. We applied the AU test to every pair of the inferred tree $T_{s}^{*}$ and pruned tree $T_{s}$ with differing topologies (i.e. TII≠0) and interpreted instability as “significant” if the AU test rejected the pruned tree by returning a *P* value of less than 0.05. As an alternative to the AU test we also tried using the SH-test (Shimodaira and Hasegawa 1999), which gave very similar results, so we decided to only use AU test *P* values for our analysis.

### Data

We analyzed datasets (Harrington et al. 2020a, b) that have previously been used in a large-scale study investigating the performance of MCMC methods for Bayesian inference (Harrington et al. 2021). This collection stems from 3 sources: (i) amniotes data containing gene sequences with small numbers of taxa (less than 50) (Brown and Thomson 2017); (ii) mtDNA sequences of all 13 protein-coding mitochondrial genes of a variety of tetrapod species giving us alignments containing between 20 and 575 sequences (Richards et al. 2018); (iii) datasets assembled by Harrington et al. (2021) from the PhyLoTA database (Sanderson et al. 2008), which contain nucleotide alignments with up to 250 sequences curated from GenBank with diverse taxon compositions and sizes. These datasets are available on DRYAD: https://doi.org/10.5061/dryad.63xsj3v9x. The computational cost of our analysis was substantial since we computed n+1 trees with 1000 bootstrap replicates for each alignment with n sequences: one full tree and for each of the n taxa in the alignment, one inferred tree. We, therefore, took a subset of 1000 alignments from the collection of datasets described above. To get this subset, we binned all alignments into bins according to quantiles of the number of sequences (15 bins) and number of sites (10 bins) and uniformly drew datasets from each bin to cover alignments of different sizes in our analysis. Because Maximum Likelihood inference is not identifiable with duplicate sequences, we deleted exact duplicates from alignments before our analyses.

We used Pythia (Haag et al. 2022) to assess the difficulty of Maximum Likelihood inference on the full alignment. Pythia uses a random forest to predict, given an alignment, whether a Maximum Likelihood inference is “easy” (best score 0.0), that is, multiple Maximum Likelihood inferences run on the same alignment are likely to converge on the same tree, or “hard” (worst score 1.0), that is, we observe a rugged tree space for this dataset. The difficulty scores of our data vary from 0 to 0.86 with a mean difficulty score of all alignments of 0.56 (Supplementary Fig. S1).

### Inferring Phylogenies

To compute stability measures, we first needed to obtain full trees $T^{*}$ and inferred trees $T_{s}^{*}$, learned from the alignment with taxon s deleted, for each taxon s in the full alignment. We inferred Maximum Likelihood trees using IQ-TREE 2.2.5 (Minh et al. 2020). Because the choice of substitution model can influence TII values (Mariadassou et al. 2012), we used ModelFinder (Kalyaanamoorthy et al. 2017) as implemented in IQ-TREE to determine the best substitution model and parameters for the full alignment and used the same model parameters for inferring trees $T_{s}^{*}$ for all s. Bootstrap support values were computed using ultrafast bootstrap (UFBoot2) (Hoang et al. 2018) with 1000 replicates.

### Workflow

**Figure 2:**
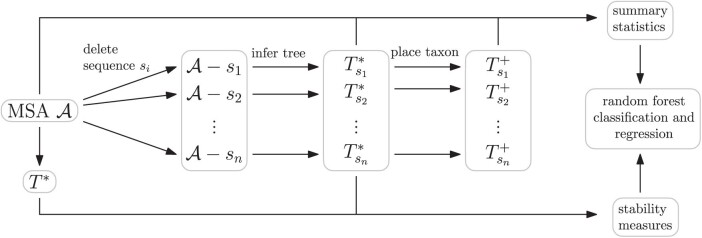
Workflow for modeling stability. We compute stability measures as described in Figure 1 and separately compute summary statistics to predict stability (without using the full tree for alignment 𝒜) using random forests.

On top of analyzing the extent to which instability occurs in empirical data, we also wanted to investigate causes of instability and see if it is possible to efficiently predict from an alignment and inferred tree whether an inference is stable for a given additional taxon. We, therefore, created a workflow (Fig. 2) to perform a stability analysis and stability prediction for our 1000 empirical datasets.

Starting with an alignment 𝒜, we inferred the full tree $T^{*}$ for this alignment and for each taxon $s_{i}$ created an alignment $𝒜-s_{i}$ that contains all sequences of the full alignment except for $s_{i}$. For each alignment $𝒜-s_{i}$ we then computed the inferred tree $T_{s_{i}}^{*}$ and also pruned $s_{i}$ from the full tree to receive the pruned tree $T_{s_{i}}$. With $T^{*}$, $T_{s_{i}}^{*}$, and $T_{s_{i}}$ we computed stability measures TII and disruption radius, as well as the distance of unstable edges to low bootstrap support edges (details in “Measuring Stability” section). To determine whether instability is significant, we performed the AU test (Shimodaira 2002) on $T_{s_{i}}$ and $T_{s_{i}}^{*}$, following Powell and Battistuzzi (2022).

We also hypothesized causes of instability and computed summary statistics as input features for random forest classification and regression to predict our stability measures (“Classification and Regression to Predict Instability” section). These summary statistics were computed using properties of the full alignment 𝒜 and inferred trees $T_{s_{i}}^{*}$. In addition, we found the best possible insertion location of taxon $s_{i}$ in the inferred tree $T_{s_{i}}^{*}$ and used properties of the resulting tree $T_{s_{i}}^{+}$ to compute summary statistics.

The entire workflow was implemented using Snakemake (Moelder et al. 2021) with coding support from ChatGPT (OpenAI 2024) and is available at https://github.com/matsengrp/phylostability. We used IQ-TREE 2.2.5 (Minh et al. 2020) for phylogenetic tree inference, PyPythia (Haag et al. 2022) for computing difficulty scores, CONSEL (Shimodaira and Hasegawa 2001) for significance testing, epa-ng (Barbera et al. 2019) for taxon placement, BioPython (Cock et al. 2009), and ete3 (Huerta-Cepas et al. 2016) for extracting summary statistics, scikit-learn (Pedregosa et al. 2011), and Optuna (Akiba et al. 2019) for random forests and hyperparameter optimization, and seaborn (Waskom 2021) and Matplotlib (Hunter 2007) for plotting.

### Summary Statistics

We used features based on summary statistics to predict whether the inferred tree $T_{s}^{*}$ is different from the pruned tree $T_{s}$, namely regression and classification random forests predicting our measures of stability. These summary statistics were calculated from the inferred tree and sequence alignment and were designed to be computed efficiently, in particular without requiring computing the full tree. Of course, if one is willing to compute the full tree as part of a stability calculation, this is a direct assay of stability rather than a prediction.

Some of our summary statistics use properties of high-quality insertion locations of the taxon s in the inferred tree $T_{s}^{*}$. We used epa-ng (Barbera et al. 2019), an evolutionary placement algorithm using a Maximum Likelihood approach to place sequences on a reference tree, to identify these insertion locations. Epa-ng returns a list of high-quality insertion locations (see details in “Spread of High-Quality Insertion Locations” section), the best of which we refer to as *the best insertion location*, giving us a tree $T_{s}^{+}$ that includes the taxon s.

#### Spread of High-Quality Insertion Locations

If there are multiple high-quality insertion locations for a taxon s in the inferred tree, it seems likely that adding the sequence of s to the inference draws these insertion locations closer together, especially when they are distant in the inferred tree. Epa-ng computes the likelihood weight ratio (LWR) of attaching the taxon s on an edge $l_{i}$, which is formally defined as $\text{LWR}\,\left(l_{i}\right)=ℒ\,\left(D|T_{s}^{*},l_{i}\right)/\sum_{j}ℒ\,\left(D|T_{s}^{*},l_{j}\right)$, where ℒ is the likelihood, D is the full alignment, and $l_{j}$ are edges in the inferred tree $T_{s}^{*}$. It returns a list of edges sorted by decreasing LWR until the sum of LWR values exceeds 0.99. We considered all edges in this list to be high-quality insertion locations.

To investigate whether the distance between high-quality insertion locations influences stability, we computed the mean and standard deviation (SD) of topological distances between the best insertion edges and used them as summary statistics (insertion distances mean/SD). These distances were normalized by dividing by the maximum distance of any two edges in the inferred tree $T_{s}^{*}$. If there was an insertion location with LWR greater than 0.99, epa-ng returns only this one location, and we set the distance between the best locations to be zero. We also used the LWR of the best insertion edge itself (LWR) as well as the number of high-quality insertion locations (*#insertion locations*) as summary statistics, as it seemed likely that high uncertainty in the placement of a taxon indicates higher instability.

We also wondered if particularly short branches around the best insertion location, which could indicate uncertainty in how clades in this region of the tree should be resolved, lead to higher instability. To address this question, we adopted the length of the branch b on which the additional taxon is inserted in $T_{s}^{*}$ (*insertion branch length*) as a summary statistic. Upon insertion of s, b is split into two branches. We added the ratio of the length of the shorter of these two branches to the length of b (*normalized distance to nearest node*) as summary statistics.

We were also curious to see if taxa that are only distantly related to the taxa in the inferred tree lead to higher instability, as adding those might result in long branch attraction (Bergsten 2005). We, therefore, added the length of the pendant branch connecting s with its parent in $T_{s}^{+}$ (*pendant length*) as a summary statistic. All branch lengths mentioned in this section were normalized by dividing by the average branch length of $T_{s}^{+}$.

#### Distance to Low Bootstrap Support Regions

We hypothesized that instability is more likely to occur when a taxon is inserted in a region of low bootstrap support in a tree. It seemed likely that especially in regions of high uncertainty, a taxon addition adds a phylogenetic signal and, therefore, influences the shape of the tree. To analyze whether this hypothesis is true, we considered the distance of the best insertion location to its closest low bootstrap support edge, normalized by the maximum distance of any edge to the insertion location (*distance to low bootstrap edge*) as a summary statistic. We identified an edge as having low bootstrap support if its bootstrap support was less than 70%.

#### Uncertainties in the Tree

To assess the difficulty of the inference on the full alignment as described in “Data” section, we computed Pythia difficulty scores on the full alignment and added them as predictors (*pythia difficulty*) to our random forests. In addition, we added the mean and standard deviation of all bootstrap support values of the inferred tree as input features for our random forests (*bootstrap mean/SD*).

#### Pairwise Sequence Distances to New Taxon

If the sequences in the inferred tree have evolved in a tree-like manner, but the sequence of the added taxon has not, Balanced Minimum Evolution trees can show instability (Cueto and Matsen 2011). We were interested to see if similar behavior can be observed for Maximum Likelihood inference. Therefore, we computed for every taxon in the tree the ratio of sequence distance to patristic distance (*sum of branch lengths*) to the added taxon s in $T_{s}^{+}$. The mean and standard deviation of these values are used as summary statistics for predicting the TII (*distance ratio mean/SD*). Our intuition was that high standard deviations of distance ratios indicate that the added sequence does not fit well into the inferred tree and did not evolve very tree-like, potentially resulting in instability.

We, in addition, wanted to focus on the difference of the evolution of the added taxon and the taxon with sequence most similar to the added taxon. If both taxa showed similar relationships to the remaining taxa, we expected the added taxon to fit well into the tree, resulting in stability. We, therefore, computed mean and standard deviation of taxon_ratio/closest_taxon_ratio (*ratio diff closest sequence mean/SD*), where taxon_ratio is the ratio of sequence to patristic distance of the added taxon and closest_taxon_ratio is this ratio for the taxon with sequence most similar to that of the added taxon.

We observed in some cases that adding a taxon resulted in moving a subtree away from the insertion location of this taxon in $T_{s}^{*}$ to a different position in the tree and designed a summary statistic to capture this. Let C and $C^{\prime }$ be sister clades in the inferred tree so that the best insertion location of s is on the branch connecting the parent of C and $C^{\prime }$ with the root of C. Let $a_{s}$ be the average pairwise distance of the added taxon to all taxa in $C^{\prime }$ and $a_{C}$ the average pairwise distance of taxa of C to $C^{\prime }$ in terms of sequence distance. We used $a_{s}/a_{C}$ as a summary statistic for predicting TII (*dist diff insertion sibling*). The further this value is from one, the more we expect the added taxon to change the local structure of the tree, increasing instability.

Whenever we required sequence distances in our computations, we used the sequence distances corrected by the substitution model chosen by ModelFinder when performing a model search with IQ-TREE on the full alignment.

#### Depth of Insertion Location in Inferred Tree

We hypothesized that a taxon inserted close to a leaf has a lower risk of leading to instability than a taxon inserted deep in the tree. To investigate whether this was true, we measured the insertion height as the patristic (sum of branch lengths) distance from the best insertion location of the added taxon s in $T_{s}^{+}$ to the nearest taxon that is not s. This value was used as a summary statistic (*insertion height*) after normalizing by dividing by the maximum possible distance between any two leaves divided by two.

#### TII s of Distance Based Inference Methods

Distance-based inference methods are computationally less expensive than Maximum Likelihood approaches, and we wondered whether different inference methods on the same data are similarly stable or unstable. We, therefore, computed Neighbor Joining (NJ) trees (Saitou and Nei 1987) on the sequence distance matrices corrected according to the substitution model chosen by ModelFinder. As we have done for Maximum Likelihood trees, we computed TII values for all taxa in all NJ trees and used normalized NJ TII values as features for predicting the stability of Maximum Likelihood trees (*NJ TII*).

### Classification and Regression to Predict Instability

We extracted summary statistics for each taxon of every alignment and used these as features to train random forests to predict and classify stability. We performed 200 trials of hyperparameter optimization, setting the following ranges for parameters for all random forests we trained: number of decision trees in [10,1000], maximum depth of a decision tree in [10,1000] (sampled from the log domain), fraction of minimum number of samples per split in [0.00001,1] (sampled from the log domain), fraction of minimum number of samples required to be a leaf node in [0.00001,1] (sampled from the log domain), and maximum number of features considered for the best split either $\sqrt{\text{num\_samples}}$ or $\log_{2}\left(\text{num\_samples}\right)$. For regression, hyperparameter optimization tested criteria for measuring the quality of a split in {squared error, absolute error, Friedman MSE, poisson}, for classification the criterion was chosen from {gini, log loss, entropy}. Our data were split into a training set (60%), validation set (20%), and test set (20%). Hyperparameter optimization was done for the validation set, and the test set was used for evaluation.

We trained 2 random forest classifiers. For a given combination of inferred tree and full sequence alignment, the input to these classifiers was the collection of summary statistics described in “Summary Statistics” section. Each classifier predicted whether a taxon addition was in 1 of 2 instability classes. For the first classifier, these classes were determined by whether the TII was 0 or not. The second classifier predicted whether we observe “significant instability,” which we define as a taxon having a non-zero TII and the corresponding inferred tree producing a significant outcome of the AU test (*P* value less than 0.05). Since the TII is 0 if and only if the disruption radius is 0, we did not train another classifier for disruption radius classification.

For training a random forest classifier it is important to have similar numbers of training samples in each class, as otherwise a predictor that always predicts the outcome to be in the larger class can perform very well without actually learning anything from the input data. In our dataset, we did, however, observe more non-zero than zero TII values, so we needed to balance the training set and testing set. For the test set, we randomly subsampled the non-zero TII class to get 2 classes of equal size. We decided to split the training set for the random forests into batches so that each batch contained all samples from the TII zero class and the same number of samples from the TII non-zero class. The samples from the larger class were drawn without replacement so that no sample of this class was used in more than one random forest. This resulted in training 7 random forests for the classifier predicting TII. After training, we evaluated each classifier on the test set and averaged probabilities over all classifiers to compute the receiver operating characteristic (ROC) curve. As there were roughly as many significantly unstable taxa as there were taxa that were not significantly unstable, we did not need to balance the datasets for random forest training to predict significant instability. We also evaluated feature importances (permutation importance (Breiman 2001)) as computed by scikit-learn, which assesses how much the model performance decreases when shuffling the values of a feature across the columns of the testing dataset. Feature importances were averaged across all classifiers and final predictions were generated using majority voting, that is, the final prediction was the class that has been predicted by the majority of classifiers.

In addition, we performed 2 random forest regressions to predict (normalized) TII and disruption radius. As for the classifiers, the input features were the summary statistics presented in “Summary Statistics” section. Because we observed that feature importances for Pythia difficulty were very high and, therefore, potentially absorbed the importance of other features, making it harder to determine which of the remaining features were good predictors, we additionally trained all types of random forests without Pythia difficulty scores. This lead to random forests with extremely high feature importance for bootstrap mean and standard deviation, so we also removed these 2 summary statistics to get better insights into feature importances.

## Results

### Instability Is Abundant

**Figure 3:**
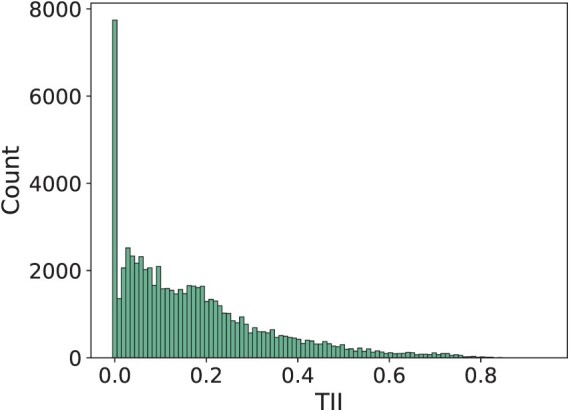
Observed normalized TII values

Instability frequently occurs in our datasets in the form of TII values greater than 0 (Fig. 3). Only 7707 out of a total of 67,709 taxa (11.38%) show stability (TII zero), that is, the addition of these taxa to the alignment results in a tree containing the tree topology of the tree inferred on the smaller alignment. The number of times we observe a given TII value declines with increasing TII, but we do observe (normalized) TII values of up to 0.96.

### Instability Is Not Always Significant

**Figure 4:**
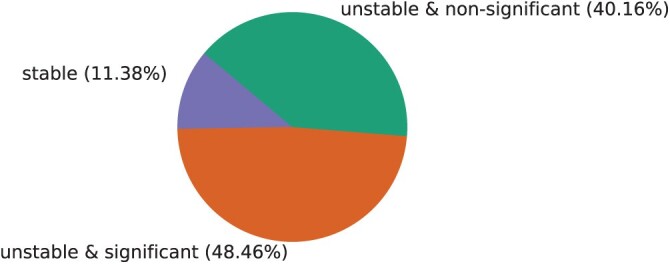
Fraction of stable (TII=0) and unstable (TII≠0) taxa together with the significance of AU test (color online)

AU test results show a significant difference between pruned and inferred trees for 48.46% of all taxa (Fig. 4), even though TII values show that instability occurs in 88.62% of our data. For 40.16% of all taxa, we observe non-significant instability, that is, non-zero TII but non-significant difference between pruned and inferred trees according to the AU test. This means that out of all unstable taxa, 54.68% are considered significantly unstable by the AU test, whereas the difference between inferred and pruned trees of the remaining 45.32% of unstable taxa is not significant.

### Instability Occurs in Low Bootstrap Regions, Not at Taxon Attachment

**Figure 5:**
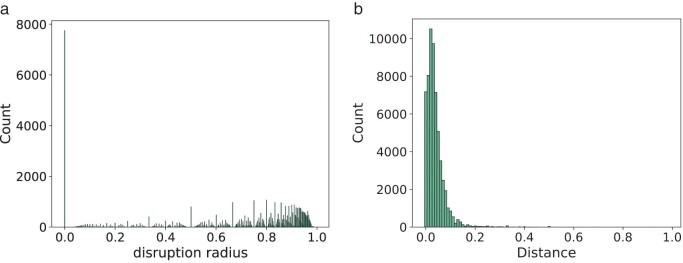
Observed locations of instability.

We often observe high disruption radii, suggesting that if instability occurs, there are often changes in the tree that are distant from the attachment of the added taxon (Fig. 5. For about half of all taxa (51.83%), we see a normalized disruption radius greater than 0.8. This indicates that locations of instability are generally not local to the attachment location. As a general trend, we observed that there are more high disruption radius taxa for large trees than for trees with fewer taxa.

We observe small distances of unstable edges to low bootstrap edges (Fig. 5, which suggests that instability almost always occurs in regions of low bootstrap support. Though there are cases where the average normalized distance between unstable edges and low bootstrap support edges is at 0.95, for 80% of our taxa this average normalized distance is less than 0.21.

### Instability Is Predictable

**Figure 6:**
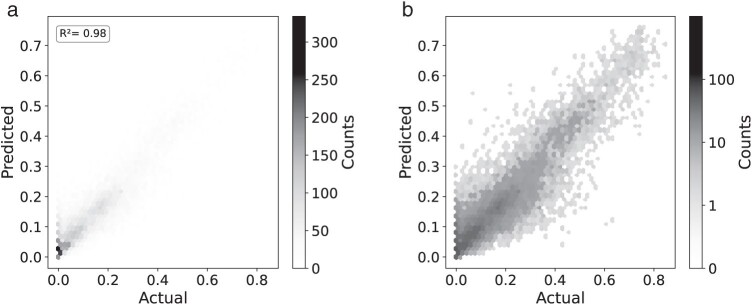
Regression results for random forest predictions of TII including mean and standard deviation of bootstrap support values of inferred tree as features. Both plots display predicted versus actual normalized TII values but in B), a $\log_{10}$ scale is used.

Random forests for TII classification, which predict TII=0 (stable) versus TII≠0 (unstable), show good discrimination. The ROC curve (Supplementary Fig. S2) of our test set has an area under the curve of 0.94. Random forest regression trained to predict normalized TII performs very well with an $R^{2}$ score of 0.98 (Fig. 6).

**Figure 7:**
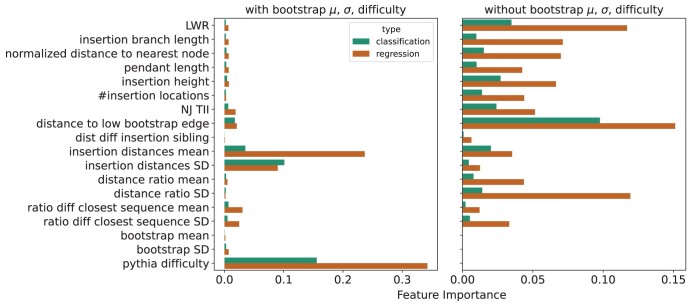
Feature importance of random forest predictions of TII including mean and standard deviation of bootstrap values and Pythia difficulty as input features on the left and excluding them on the right. For each pair of bars, the top bar shows feature importances for TII classification (TII zero vs. non-zero) and the bottom bar shows feature importances for TII regression. The higher its feature importance, the more influence a feature has on the final prediction. Feature importances sum up to one for each random forest.

We use feature importance to evaluate which of our summary statistics have the biggest influence on whether the random forest predicts stability or instability. Pythia difficulty has the highest importance for regression and classification, followed by the distance between high-quality insertion locations as determined by epa-ng (Fig. 7, left panel). We were interested in seeing whether the Pythia difficulty scores absorbed the importance of other features. After training a random forest without difficulty scores as predictors, bootstrap mean and standard deviation received much higher feature importance than all other features, so we trained a random forest without Pythia difficulty and mean and standard deviation of bootstrap support. Excluding these summary statistics slightly worsens the quality of predictions, with the area under the ROC curve for TII classification dropping to 0.86 (Supplementary Fig. S2) and the $R^{2}$ score dropping to 0.94 (Supplementary Fig. S3).

Removing bootstrap values of the inferred trees and difficulty scores of the full alignment as predictors results in a classifier that gives the highest importance to the distance of the insertion location to its closest low bootstrap support edge (Fig. 7, right panel). This suggests that the proximity of insertion locations to regions of uncertainty influences how much a tree changes when a taxon is added to the inference. The mean normalized distance of insertion locations to low bootstrap support edge for unstable taxa is 0.13, whereas it is 0.26 for stable taxa, indicating that insertion locations in regions of high uncertainty in a tree lead to instability.

From the regression, we also observe high importance for the distance of the insertion location to its closest low bootstrap support edge as well as for likelihood weight ratio and the standard deviation of the ratios of sequence to patristic distance of the added taxon to all taxa in the inferred tree (Fig. 7). This supports the hypothesis that insertion locations close to regions of high uncertainty in the tree influence instability, as does the existence of multiple high-quality placement locations (*LWR*) and the addition of a taxon evolving in a non-tree-like manner (*distance ratio SD*).

Disruption radius regression results show a high correlation with an $R^{2}$ score of 0.94 (Supplementary Fig. S4). Feature importances show that Pythia difficulty and distances between high-quality insertion locations have the biggest influence on stability predictions (Supplementary Fig. S5, left panel). Removing bootstrap support values and Pythia difficulty for disruption radius predictions resulted in a slightly worse predictor with an $R^{2}$ score of 0.9 (Supplementary Fig. S4), and insertion height and distance of the insertion location to the nearest low bootstrap support edge had the highest feature importance (Supplementary Fig. S5, right panel).

The random forest classifier predicting significant instability, that is, whether the *P* value of the AU test is less than 0.05 and the TII is non-zero, performs slightly worse than our other predictors with an area under the ROC curve of 0.86 (Supplementary Fig. S6). Feature importances show the high importance of the length of the pendant edge leading to the added taxon when placed in the inferred tree (Supplementary Fig. S7). Removing bootstrap support values for predicting significant instability resulted in the same observations and led to a very similar $R^{2}$ score of 0.83 and a similar pattern of feature importances. This shows that for our data different features are of high importance when predicting significant instability compared to predicting just instability (TII≠0). Re-training our models reliably showed the same patterns of feature importances, suggesting that the most influential features for the two predictors do in fact differ.

## Discussion

In this article, we performed a stability analysis investigating how much a phylogenetic tree topology changes when new sequences are added to an inference. Our results showed frequent instability, with 88.62% of taxa having a non-zero TII and disruption radius. The AU test revealed that this difference between inferred and pruned trees is not always significant. This suggests that inserting a taxon at the best position in an already inferred tree often gives trees that are of similar quality to the tree inferred on the full alignment. High Pythia difficulty scores support this, as they indicate that there are multiple high-quality trees for the full alignment. While we focused on the addition of a single taxon, future work could include adding multiple taxa to an inference and analyzing how many taxa can be added before the tree changes significantly. That the choice of substitution model used for inference influences the TII value (Mariadassou et al. 2012) also needs to be taken into account when developing online methods for phylogenetic inference.

If instability occurred, it was often not local to the attachment location of the added taxon, which we saw as more than 50% of taxa in our datasets had a normalized disruption radius greater than 0.8. We observed that unstable edges were often close to edges with low bootstrap support. This observation, together with our Pythia difficulty and AU test results, suggests that changes in trees upon taxon addition can be the result of multiple trees being similarly good according to the Maximum Likelihood criterion. If this is the case, the addition of a taxon can change the topology of the backbone tree to a tree topology that also has a high likelihood.

Using random forest regression and classification, we could reliably predict instability. We found that insertion locations in low bootstrap support regions and Pythia difficulty scores were good predictors for instability. For TII random forest regression we also observed that uncertainties in the placement of the added taxon in the inferred tree with epa-ng are influential predictors of instability. The random forest classifier trained to predict significant instability, that is, non-zero TII and AU test *P* value less than 0.05, performed marginally worse than TII classification. A reason for this worse performance of predictions of significant instability could be that the random forests also need to capture uncertainties in the inferred tree, making predictions more complex. Feature importances were different for significant instability compared to instability, suggesting that these 2 cases may have different causes.

We additionally found that adding the mean and standard deviation of bootstrap values of the inferred tree as well as Pythia difficulty scores resulted in better predictions. These features also received high importance in most random forests. This again shows that uncertainties in inferred trees are a major driver of instability. Only for predictions of significant instability, bootstrap and difficulty have similar importance to most other features. These measures of uncertainty performed well at predicting the extent to which we observe instability in our random forest regressions, but they are less good at predicting whether instability is significant or not.

For our analysis, we used empirical datasets, as we wanted to see the effect that taxon additions have in real-world examples, where online algorithms would actually be used. There are, however, many different parameter regimes in phylogenetics, and in our study we focused on gene tree inference using Maximum Likelihood methods. Viral datasets like SARS-CoV-2, for example, are usually densely sampled and may behave differently. In addition, different inference methods may show differences in stability. We could observe the low feature importance of the Neighbor Joining TII for Maximum Likelihood TII in our random forests, indicating that Neighbor Joining instability does not necessarily imply Maximum Likelihood instability. It seems likely that stability for Bayesian inference behaves more similar to Maximum Likelihood inference, as Bayesian inferences incorporate the likelihood. Further studies are however necessary to confirm this, which in particular would involve developing a way to compare 2 posterior distributions of trees: before and after taxon addition.

Our approach to computing stability measures for every taxon of an alignment has previously been proposed by Powell and Battistuzzi (2022), where the authors suggest analyzing the effect of taxon sampling on phylogenetic inference. We extended this idea by training random forests to predict stability and applied our framework to empirical data and could thereby identify causes for instability.

It has previously been suggested that instability might be local to the attachment location of the added taxon (Truszkowski et al. 2023). We observed the opposite. In our analysis, instability often occurred distant from the attachment location, but close to regions of low bootstrap support. This behavior might not generalize to all settings, as it might depend on data and inference method.

The idea of instability being local to the insertion location has been used for some online inference methods, where trees are first updated around the insertion location before global updates are performed (Bouckaert et al. 2022). Other methods perform updates throughout the tree (Ye et al. 2022) without focusing on specific regions of the tree. Our results suggest that an alternative, potentially more efficient approach, is to update the tree after insertion in regions of high uncertainty. As our results are limited to Maximum Likelihood inference on gene trees, it remains to be tested whether this would lead to better performance of online inference methods in general.

In summary, our study showed that we can predict instability from our summary statistics. However, with an area under the ROC curve of 0.94 for our TII classifier, the predictions were not correct for all taxon additions, suggesting that more work would be needed if this framework was to be used as a basis for online algorithms. It seems feasible that a pipeline of placing taxa in a tree, efficiently predicting stability, and updating the tree after placement based on those predictions, could result in an efficient and accurate online algorithm for phylogenetic inference.

## Acknowledgements

This work was supported through the US National Institutes of Health grant AI162611. Scientific Computing Infrastructure at Fred Hutch was funded by ORIP grant S10OD028685. Dr Matsen is an Investigator of the Howard Hughes Medical Institute.

## Supplementary Materials

Data available from the Dryad Digital Repository: https://doi.org/10.5061/dryad.63xsj3v9x.

## Conflict of Interest

None declared.

## Data Availability

Supplementary material, including data files and/or online-only appendices, can be found in the Dryad data repository (https://doi.org/10.5061/dryad.63xsj3v9x).
